# Supplementary material for: Ubiquitination Is a Novel Post-Translational Modification of VMP1 in Autophagy of Human Tumor Cells
Source: Int J Mol Sci. 2023 Aug 19;24(16):12981. doi: 10.3390/ijms241612981 (PMC10455450; doi:10.3390/ijms241612981)
Supplement: Supplementary file 1 [file ijms-24-12981-s001.zip › ijms-2491340-supplementary.pdf]

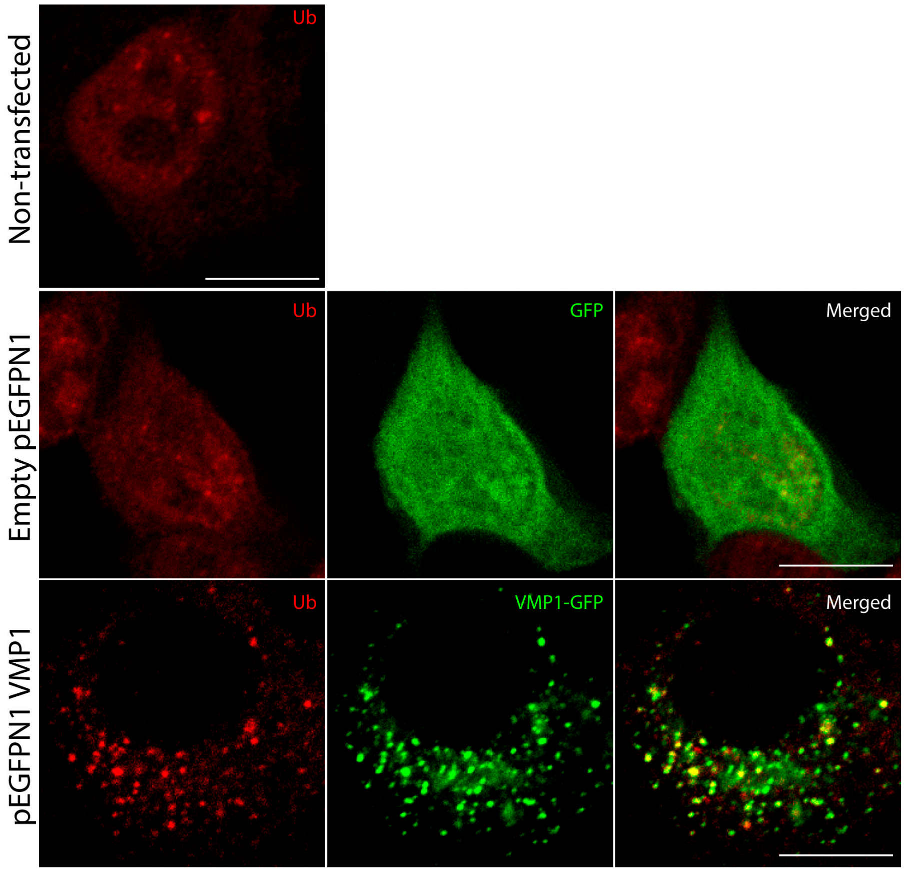

Figure S1. Ubiquitin recruitment in PANC-1 cells. PANC-1 cells expressing Empty pEGFPN1 or pEGFPN1 VMP1, or non-transfected PANC-1 cells, were immunolabeled with anti-ubiquitin. Scale bars: 10 μm.

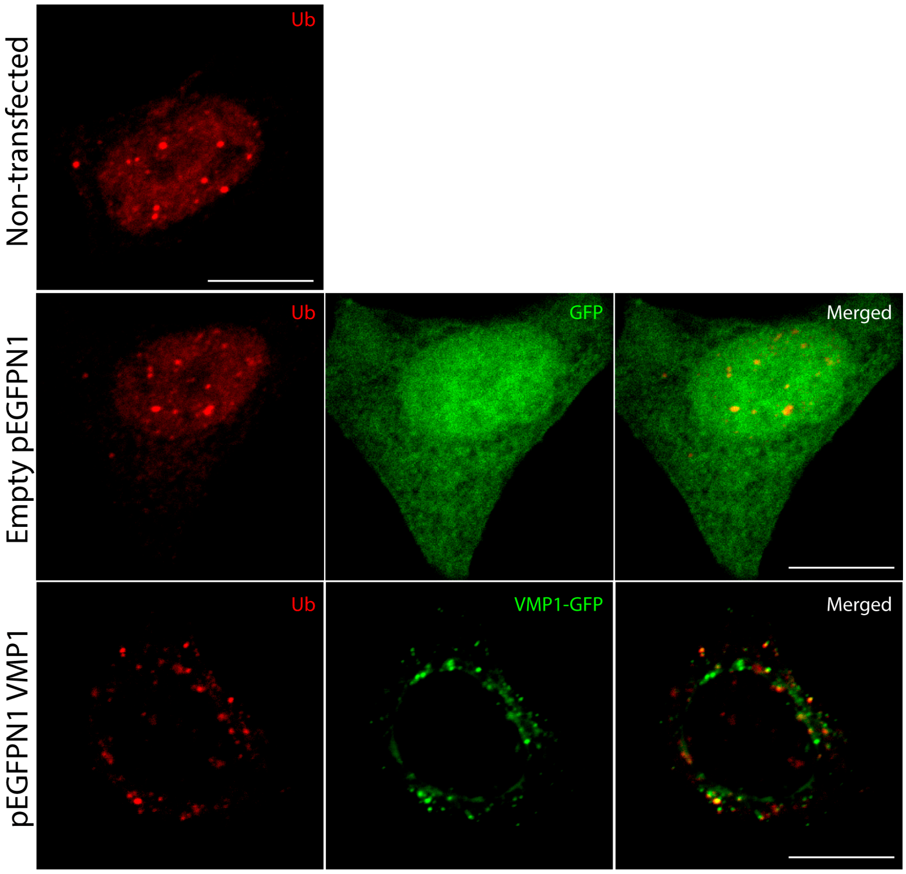

Figure S2. Ubiquitin recruitment in MCF-7 cells. MCF-7 cells expressing Empty pEGFPN1 or pEGFPN1 VMP1, or non-transfected MCF-7 cells, were immunolabeled with anti-ubiquitin. Scale bars: 10 μm.

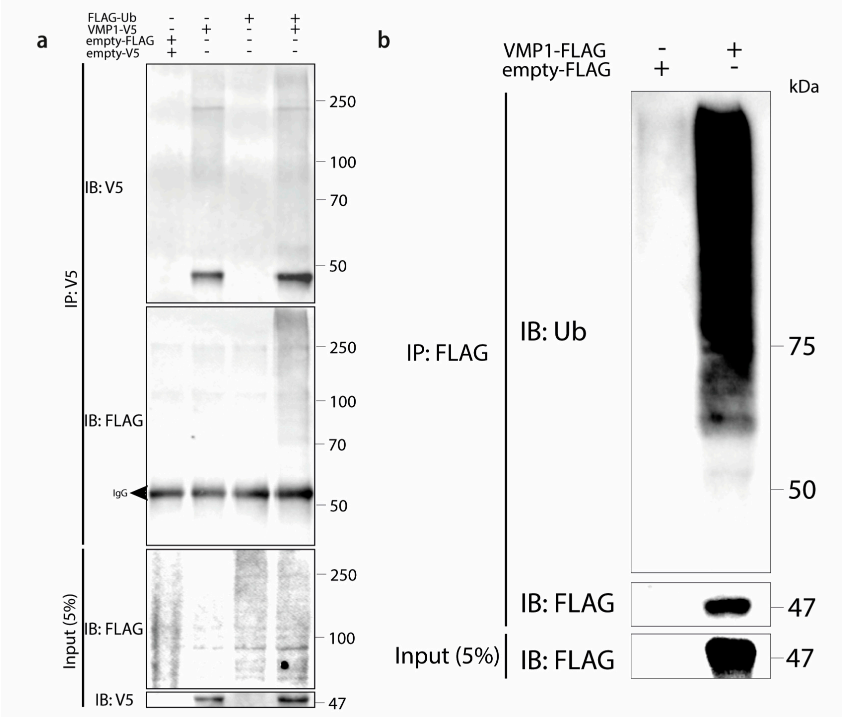

Figure S3. Immunoprecipitation of VMP1 and Ubiquitin. **a.** Lysates from HEK293T cells transfected with VMP1–V5, FLAG–Ub, and the combination of both were immunoprecipitated with anti-V5 magnetic beads and immunoblotted with anti-FLAG. Several bands over VMP1–V5's molecular weight (47.5 kDa) appeared in the eluate of co-transfected cells, indicating VMP1 ubiquitination. **b.** Lysates from HEK293T cells transfected with VMP1–FLAG were immunoprecipitated with anti-FLAG magnetic beads and immunoblotted with anti-Ub. Several bands over VMP1–FLAG's molecular weight (47 kDa) appeared in the eluate, indicating VMP1 ubiquitination.

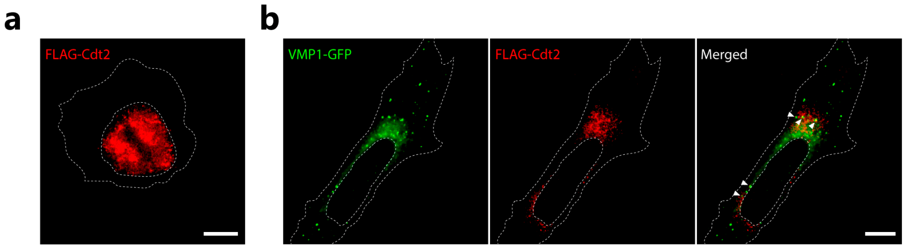

Figure S4. FLAG-Cdt2 translocation. (**a**) HeLa cells were transfected with FLAG-Cdt2 and immunolabeled with anti-FLAG. Scale Bar: 10 μm. (**b**) HeLa cells were transfected with VMP1-GFP and FLAG-Cdt2 and immunolabeled with anti-FLAG. Scale Bar: 10 μm. The images are representative of three independent experiments.
